# Оценка абсорбции левотироксина натрия в реальной клинической практике путем проведения перорального теста с одномоментным приемом болюсной дозы

**DOI:** 10.14341/probl13665

**Published:** 2026-01-18

**Authors:** Е. А. Трошина, Н. В. Мазурина, М. Х. Боташева, Н. М. Платонова, А. П. Першина-Милютина, И. Р. Гасымова

**Affiliations:** Национальный медицинский исследовательский центр эндокринологии им. академика И.И. ДедоваРоссия; Endocrinology Research CentreRussian Federation

**Keywords:** гипотиреоз, левотироксин натрия, мальабсорбция, тест на всасывание левотироксина натрия, hypothyroidism, levothyroxine sodium, malabsorption, levothyroxine sodium absorption test

## Abstract

**ОБОСНОВАНИЕ:**

ОБОСНОВАНИЕ. Результаты заместительной терапии гормонами щитовидной железы остаются неудовлетворительными в 30–50% случаев. Для дифференциальной диагностики истинной мальабсорбции левотироксина натрия и псевдомальабсорбции вследствие низкой комплаентности предлагается несколько вариантов теста по оценке всасывания препарата с различными критериями оценки.

**ЦЕЛЬ:**

ЦЕЛЬ. Определить критерии нормальной абсорбции левотироксина натрия при проведении перорального теста с одномоментным приемом болюсной дозы 600 мкг.

**МАТЕРИАЛЫ И МЕТОДЫ:**

МАТЕРИАЛЫ И МЕТОДЫ. В исследовании приняли участие 20 здоровых добровольцев обоих полов в возрасте от 18 до 35 лет с нормальной массой тела. Болюсная доза левотироксина натрия составляла 600 мкг. Образцы крови для определения концентрации свободного тироксина (свТ4) в сыворотке крови были взяты натощак, через 1 час, через 2, 3, 4, 6 часов после приема препарата.

**РЕЗУЛЬТАТЫ:**

РЕЗУЛЬТАТЫ. После приема левотироксина натрия в дозе 600 мкг максимальный уровень свТ4 отмечался через 2 часа — 21,00 пмоль/л [19,20; 23,16]. Минимальное увеличение концентрации свТ4 через 2 часа после приема левотироксина натрия в дозе 600 мкг составило — 18,0%, максимальное — 91,1%.

**ЗАКЛЮЧЕНИЕ:**

ЗАКЛЮЧЕНИЕ. Для оценки всасывания левотироксина натрия рекомендуется проведение теста — однократный пероральный прием левотироксина натрия в дозе 600 мкг. Всасывание левотироксина натрия может быть расценено как нормальное, если увеличение концентрации свТ4 через 2, 3 или 4 часа после приема болюсной дозы составляет не менее 18%.

## Abstract (en)

**BACKGROUND:**

BACKGROUND: Thyroid hormone replacement therapy results remain unsatisfactory in 30-50% of cases. To differentiate between true levothyroxine sodium malabsorption and pseudomalabsorption due to poor compliance, several absorption assessment tests with different evaluation criteria are proposed.

**AIM:**

AIM: To determine the criteria for normal absorption of levothyroxine sodium when performing an oral test with a single bolus dose of 600 mсg.

**MATERIALS AND METHODS:**

MATERIALS AND METHODS: The study involved 20 healthy volunteers of both sexes, aged 18 to 35 years, with normal body weight. The bolus dose of levothyroxine sodium was 600 mсg. Blood samples for determination of free thyroxine (fT4) concentration in serum were taken on an empty stomach, 1 hour, 2, 3, 4, 6 hours after taking the drug.

**RESULTS:**

RESULTS: After taking levothyroxine sodium at a dose of 600 mcg, the maximum level of free T4 was observed after 2 hours — 21,00 pmol/l [19,20; 23,16]. The minimum increase in the concentration of free T4 2 hours after taking levothyroxine sodium at a dose of 600 mcg was — 18,0%, the maximum — 91,1%.

**CONCLUSION:**

CONCLUSION: To assess the absorption of levothyroxine sodium, a single oral dose of 600 mcg of levothyroxine sodium is recommended. Absorption of levothyroxine sodium can be considered normal if the increase in free T4 concentration 2, 3, or 4 hours after the bolus dose is at least 18%.

## ОБОСНОВАНИЕ

Гипотиреоз — одно из самых распространенных заболеваний эндокринной системы, а левотироксин натрия входит в десятку наиболее часто назначаемых в мире препаратов. Коммерческий препарат синтетического левотироксина натрия появился в 1955 г. и быстро завоевал популярность благодаря безопасности и точности дозирования. Дальнейшие исследования привели к тому, что к 80-м годам XX в. синтетический препарат практически полностью вытеснил натуральные экстракты, а левотироксин натрия стал абсолютным стандартом лечения гипотиреоза [1]. Несмотря на доступность заместительной терапии гормонами щитовидной железы, в клинической практике все еще существуют значительные проблемы, а результаты лечения остаются неудовлетворительными в 30–50% случаев [2]. Наиболее частыми причинами неудовлетворительной компенсации гипотиреоза являются плохая приверженность пациентов к терапии (пропуск приема препарата, нарушения режима приема, перерывы в лечении) и нарушение всасывания левотироксина натрия на фоне заболеваний желудочно-кишечного тракта и приема лекарственных средств, влияющих на его биодоступность [3][4].

Левотироксин натрия является препаратом выбора для заместительной терапии гипотиреоза в силу его эффективности, длительного опыта применения, высокой биодоступности, благоприятного профиля нежелательных явлений и простоты приема. После растворения и ионизации таблеток левотироксина в кислой среде желудка препарат всасывается в тонком кишечнике (двенадцатиперстной кишке, тощей кишке). Концентрация тироксина в плазме крови повышается в течение первых 60–90 минут и достигает пика через 2 часа после приема таблетированного левотироксина. Для достижения компенсации требуется от 1,6 до 1,8 мкг левотироксина натрия на кг массы тела в сутки [5]. Этого достаточно для поддержания нормальной концентрации ТТГ при первичном гипотиреозе или концентрации свТ4 в пределах нормы при центральном гипотиреозе. Однако у некоторых пациентов, несмотря на увеличение дозы левотироксина до 1,9 мкг/кг/день, достичь эутиреоза не удается. К факторам, препятствующим достижению целевых показателей на фоне заместительной терапии левотироксином натрия, относятся: прием неадекватной дозы препарата, прием лекарственных средств или биологически активных добавок, влияющих на всасывание или фармакокинетику левотироксина, нарушения режима приема препарата — псевдомальабсорбция. Заболевания желудочно-кишечного тракта (гастрит, ассоциированный с Helicobacter pylori, аутоиммунный гастрит, целиакия), гастрэктомия, бариатрическая хирургия — могут быть причиной истинной мальабсорбции [6–7]. В клинической практике гипотиреоз, рефрактерный к лечению в связи с истинной мальабсорбцией, встречается значительно реже, чем псевдомальабсорбция.

Лабораторным критерием эффективности заместительной терапии первичного гипотиреоза является нормальный уровень ТТГ, целевые значения которого зависят от ряда факторов (этиология гипотиреоза, возраст пациента, наличие сопутствующих заболеваний). Так, по данным, полученным в ходе The Colorado thyroid disease prevalence study (2000 г.), среди участников исследования, принимавших таблетированные препараты для лечения заболеваний щитовидной железы (n=1525), только в 60% случаев концентрация ТТГ в сыворотке крови соответствовала референсным значениям [8]. В рамках другого американского исследования (Somwaru L. et al.) была проведена оценка эффективности заместительной терапии левотироксином натрия среди лиц в возрасте 65 лет и старше [9]. В этой когорте пациентов, получавших таблетированный левотироксин, только 43% имели нормальный уровень ТТГ, у 41% ТТГ был низким, у 16% — высоким.

Для дифференциальной диагностики истинной мальабсорбции левотироксина натрия и псевдомальабсорбции вследствие низкой комплаентности в течение последних 10 лет были предложены несколько вариантов проведения теста по оценке всасывания препарата [10–15]. В рамках различных протоколов теста рассматриваются как различные нагрузочные пероральные дозы левотироксина натрия (от 400 до 2500 мкг), так и различные критерии нормальной абсорбции (абсолютные или относительные значения свТ4, площадь под кривой свТ4, количество всосавшегося левотироксина, рассчитанного при помощи формулы). Отсутствие стандартной методики является одной из причин редкого использования пробы по оценке всасывания левотироксина натрия в рутинной практике.

Ответ на вопрос о причине плохой компенсации гипотиреоза дает возможность определить способ решения проблемы, поэтому мы выбрали наиболее удобную с практической точки модификацию пробы для оценки всасывания левотироксина натрия и провели ее в группе здоровых добровольцев.

## ЦЕЛЬ ИССЛЕДОВАНИЯ

Определить критерии нормальной абсорбции левотироксина натрия при проведении перорального теста с одномоментным приемом болюсной дозы 600 мкг.

## МАТЕРИАЛЫ И МЕТОДЫ

## Характеристика группы здоровых добровольцев

В исследовании приняли участие 20 здоровых добровольцев обоих полов в возрасте от 18 до 35 лет с нормальной массой тела. Медиана возраста составила 24 года. Никто из добровольцев не имел заболеваний щитовидной железы, хронических заболеваний желудочно-кишечного тракта, пищевой аллергии и ограничений в питании. Проведены антропометрические измерения, определены рост и вес каждого из добровольцев. Медиана роста составила 168 см, медиана массы тела — 65 кг. Подробная характеристика обследованной группы добровольцев представлена в таблице 1.

**Table:** Таблица 1. Общая характеристика группы

Признак	Me [ Q1; Q3] N=20
Возраст, лет	24 [ 24; 25]
Пол	Мужской	1 (5%)
Женский	19 (95%)
Рост, см	168 [ 163; 171]
Вес, кг	65 [ 58; 74]

## Место проведения исследования

ГНЦ РФ ФГБУ «НМИЦ эндокринологии им. академика И.И. Дедова» Минздрава России.

## Дизайн исследования

До начала исследования добровольцы были проинформированы о порядке проведения и целях исследования, всеми было подписано информированное добровольное согласие на участие в исследовании.

Перед проведением теста всем добровольцам было рекомендовано воздержаться от приема пищи в течение 8–10 часов. Никто из добровольцев не принимал лекарственные средства, влияющие на всасывание тироксина — ингибиторы протонной помпы, препараты железа, препараты кальция, слабительные. Длительность проведения теста составляла 6 часов. Доза левотироксина натрия, принятая добровольцами одномоментно в виде таблеток, составляла 600 мкг. Образцы крови для определения концентрации свободного тироксина (свТ4) в сыворотке крови были взяты натощак, через 1 час, через 2, 3, 4, 6 часов после приема болюсной дозы левотироксина натрия. Никто из участников исследования не сообщил о возникновении нежелательных явлений в ходе проведения пробы и в течение 24 часов после пробы.

## Методы

Лабораторная диагностика проводилась в клинико-диагностической лаборатории ФГБУ «НМИЦ эндокринологии им. академика И.И. Дедова» Минздрава России. Концентрацию свободного тироксина в сыворотке крови определяли с помощью стандартных наборов на анализаторе Architect 2000 (Abbot Diagnostics, США). Референсный интервал для свТ4 в плазме крови 9,00–19,00 пмоль/л.

## Статистический анализ

Статистический анализ данных выполнен с помощью пакета прикладных программ STATISTICA 13.3.0 (TIBCO Software Inc., США). Количественные данные представлены в виде медианы, значения первого и третьего квартилей — Me [ Q1; Q3], минимальных и максимальных значений (Min, Max), качественных — в виде абсолютных и относительных частот (n (%)). Сравнение количественных данных при последовательных измерениях выполнялось с помощью критерия Вилкоксона (Wilcoxon). Статистически значимым признавали уровень ошибки первого рода менее 0,05. Для нивелирования проблем множественных сравнений применялась поправка Бонферрони. После применения поправки, значения р в диапазоне между рассчитанными и 0,05 интерпретировались как статистическая тенденция.

## Этическая экспертиза

Протокол исследования был одобрен локальным этическим комитетом ФГБУ «НМИЦ эндокринологии им. академика И.И. Дедова» Минздрава России. Выписка из протокола №18 от 09.10.2024 г.

## РЕЗУЛЬТАТЫ

В базальной точке до приема болюсной дозы левотироксина натрия медиана концентрации свТ4 составляла 12,70 пмоль/л [ 12,25; 13,75] (рис. 1). После приема левотироксина натрия в дозе 600 мкг максимальный уровень свободного свТ4 отмечался через 2 часа — 21,00 пмоль/л [ 19,20; 23,16]. В дальнейшем концентрация свТ4 постепенно снижалась, однако спустя 6 часов уровень свТ4 сохранялся на значительно более высоком уровне, чем в базальной точке — 20,00 пмоль/л [ 18,85; 20,75].

**Figure:**
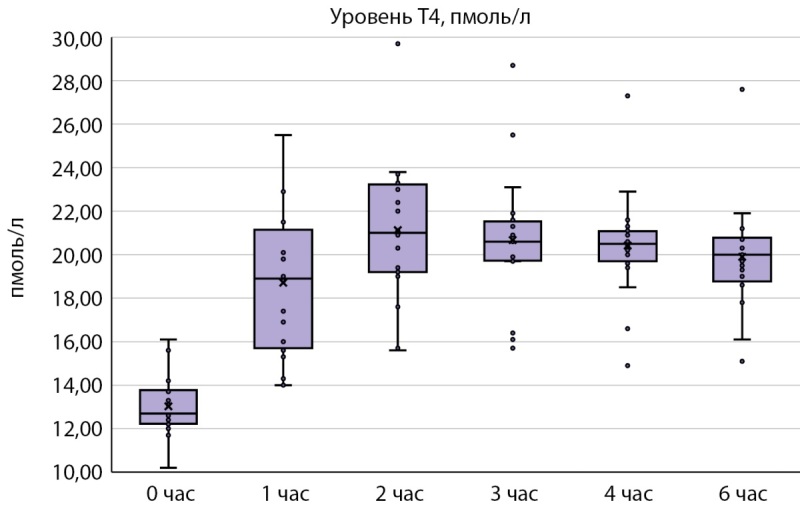
Рисунок 1. Концентрация свТ4 в сыворотке крови в ходе пробы по оценке всасывания левотироксина натрия. Примечание. Концентрация свТ4 в пмоль/л, данные представлены в виде Me [ Q1; Q3], минимальных и максимальных значений (Min, Max).

Как видно из представленных данных (табл. 2), наибольшая разница в концентрации свТ4 отмечалась при сравнении базального уровня и уровня через 2 часа после приема левотироксина натрия: абсолютное значение увеличения уровня свТ4 составило 8,60 пмоль/л [ 5,70; 10,40], относительное значение — 67,90% [ 47,40; 82,70]. Минимальное увеличение концентрации свТ4 через 2 часа после приема левотироксина натрия в дозе 600 мкг составило — 18,0%, максимальное — 91,1% (табл. 3).

**Table:** Таблица 2. Изменения концентрации свТ4 в различные временные точки при проведении пробы по оценке всасывания левотироксина натрия Примечание. Различия между уровнями свТ4 в пмоль/л, данные представлены в виде Me [ Q1; Q3].

Абсолютный прирост/снижение концентрации свТ4	Me [ Q1; Q3] пмоль/л	p
Δ свT4 0-1	5,55 [ 3,30; 7,85]	p01-12=0,011 p12-23=0,001 p23-34=0,514 p34-46=0,550
Δ свT4 0-2	8,60 [ 5,70; 10,40]
Δ свT4 1-2	2,60 [ 1,00; 3,50]
Δ свT4 2-3	-0,40 [ -1,93; 0,60]
Δ свT4 3-4	-0,30 [ -1,15; 0,45]
Δ свT4 4-6	-0,55 [ -0,90; 0,15]

**Table:** Таблица 3. Относительные изменения концентрации свТ4 в различные временные точки при проведении пробы по оценке всасывания левотироксина натрия Примечание. Относительная разница между уровнями свТ4 (%), данные представлены в виде Me [ Q1; Q3].

Относительный прирост/снижение концентрации свТ4	Me [ Q1; Q3] %	p
Δ свT4 0-1	43,2 [ 26,4; 60,7]	p012=0,002 p123<0,001 p234=0,668 p346=0,469
Δ свT4 0-2	67,9 [ 47,4; 82,7]
Δ свT4 1-2	14,3 [ 5,2; 21,55]
Δ свT4 2-3	-1,95 [ -8,5; 3,55]
Δ свT4 3-4	-1,5 [ -5,0; 2,5]
Δ свT4 4-6	-3,05 [ -4,35; 0,8]

## ОБСУЖДЕНИЕ

В нашем исследовании после перорального приема левотироксина натрия в болюсной дозе 600 мкг пик концентрации свТ4 в плазме крови был достигнут через 2 часа после приема препарата, что соответствует данным, полученным при изучении фармакокинетики препарата после перорального приема [4–6].

При проведении теста была использована нагрузочная доза левотироксина натрия 600 мкг. Выбор именно такой дозы был обоснован несколькими аргументами. Во-первых, для пациента практически с любой массой тела доза левотироксина в этом случае превысит 3 мкг/кг, и можно расценивать ее как супрафизиологическую. Следовательно, если не нарушена абсорбция препарата, уровень свТ4 должен значимо повыситься. Во-вторых, при приеме левотироксина в дозе 1000 мкг, которая использовалась в ряде исследований, у некоторой части пациентов отмечались нежелательные явления (учащенное сердцебиение, аритмия) [10][12][16–18]. Следует еще раз отметить, что в нашем исследовании после приема 600 мкг в течение 24-часового наблюдения испытуемые не сообщали о каких-либо побочных эффектах.

Для исследований фармакокинетики и биодоступности левотироксина натрия Управление по контролю за продуктами питания и лекарствами США (FDA) также рекомендует использовать дозы, не превышающие 600 мкг [19]. В документе FDA подчеркивается, что доза левотироксина натрия 600 мкг позволяет достичь очевидного пика концентрации свТ4 по сравнению с исходным уровнем.

Результаты изучения фармакокинетики левотироксина натрия как в таблетированной, так и в жидкой форме, показали, что максимальная амплитуда изменения концентрации свТ4 после приема 200 мкг не превышает 45% [6][20]. При приеме 600 мкг левотироксина натрия в таблетированной форме в нашем исследовании максимальное увеличение концентрации свТ4 составило 91,1% (медиана 67,9%). При этом у 17 из 20 испытуемых в одной или нескольких пробах крови концентрация свТ4 превысила верхнее значение референсного интервала.

Одним из главных вопросов, возникающих при назначении супрафизиологической дозы левотироксина, является вопрос безопасности. Интересно, что ранее с целью повышения приверженности пациентов терапии предлагался однократный прием недельной дозы левотироксина натрия [21]. Часть пациентов предпочла именно такую схему лечения, и нежелательных явлений при этом не отмечалось. Самому старшему пациенту, который вошел в группу получавших высокую дозу левотироксина 1 раз в неделю, было 88 лет. По данным метаанализа Dutta D. и соавт. [22], однократный прием болюсной дозы левотироксина 1 раз в неделю, по сравнению со стандартным ежедневным приемом препарата, является менее эффективным с точки зрения компенсации гипотиреоза и сопровождается повышением уровня свТ4 выше нормального референсного интервала через 2–4 часа после приема препарата. В 4 рандомизированных контролируемых исследованиях продолжительностью не менее 6 недель приняли участие 294 пациента, из которых 155 получали высокую дозу левотироксина раз в неделю. Ни в одном из исследований не было зафиксировано значимых побочных явлений со стороны сердечно-сосудистой системы. Таким образом, можно утверждать, что однократный прием 600 мкг левотироксина натрия (что сопоставимо с общей дозой заместительной терапии за 7 дней) является безопасным. Безусловно, даже однократное назначение высокой дозы левотироксина противопоказано пациентам с нестабильной стенокардией, критической ишемией миокарда, требующей интервенционных мероприятий, пациентам с нарушениями ритма сердца.

При проведении теста в группе здоровых добровольцев, то есть в ситуации, когда заведомо исключены факторы, влияющие на всасывание левотироксина натрия, основной задачей было определить критерии, которые могут быть использованы для интерпретации результатов в клинической практике. Наиболее удобными для практикующего врача являются определение относительного или абсолютного увеличения концентрации свТ4 в крови, поскольку для этого не требуется проведения сложных расчетов и измерения нескольких параметров.

Так, например, использование формулы для расчета процента абсорбированного препарата предполагает не только определение уровня общего Т4 в мкг/дл (при расчетах используется абсолютное увеличение концентрации общего Т4), но и вычисление объема распределения (0,442 х индекс массы тела) [11][14]. По данным авторов этой методики, нормальная абсорбция левотироксина натрия при пероральном приеме должна составить не менее 60%.

Определение площади под кривой концентрации свТ4 (AUC свT4) является более точным инструментом для оценки количества всосавшегося левотироксина натрия, поскольку зависит исключительно от степени всасывания и клиренса препарата, однако и для вычисления AUC свT4 требуется использование специальной формулы: либо определяется интеграл от концентрации лекарственного средства в плазме крови в зависимости от времени, либо концентрация лекарственного средства измеряется в определенные моменты времени, а для оценки AUC используется правило трапеций.

Наиболее удобным и надежным представляется определение максимальной концентрации свТ4 в плазме крови после назначения левотироксина натрия и сопоставление ее с исходным уровнем. Sun G. и соавт. [12] подтвердили, что пиковые значения свТ4 и общего Т4 в ходе проведения пробы коррелируют в достаточной степени (R=0,88). Это позволяет определять только свТ4 и делает возможным проведение теста в большинстве учреждений. Очевидно, что исходная концентрация свТ4 будет варьировать в зависимости от степени компенсации гипотиреоза: от практически нормальной у пациентов с уровнем ТТГ до 10 мЕд/л до значительно сниженной на фоне длительного перерыва в приеме левотироксина. Именно поэтому для интерпретации пробы важно оценивать не максимальную концентрацию свТ4, а ее динамику на фоне приема болюсной дозы. Прирост концентрации свТ4 может быть оценен как в абсолютном значении (пмоль/л), так и в относительном значении (%).

Авторы исследований, опубликованных в 2024–2025 гг., также предлагают оценивать абсорбцию тироксина на основании прироста концентрации свТ4, выраженной в абсолютном значении [23] или в процентах [24]. Caron P. и соавт. в ретроспективном исследовании оценивали и процент всосавшегося левотироксина с использованием формулы и динамику концентрации общего Т4 и свТ4 [10]. В рамках обсуждения полученных результатов авторы предлагают все-таки использовать для интерпретации теста концентрацию свТ4 и ограничить продолжительность теста 6 часами, а забор крови проводить через 3 и 4 часа после приема левотироксина.

При анализе динамики концентрации свТ4 в нашем исследовании (рис. 1) видно, что уровень свТ4 начинает снижаться в интервале от 2 до 3 часов после приема левотироксина per os. Только у 3 из 20 испытуемых отмечалось незначительное увеличение свТ4 в этом временном интервале, а также в интервале с 3 до 4 часов. Учитывая полученные данные, целесообразно проводить забор крови через 2 часа от начала пробы и расценивать свТ4 в этой точке как максимальный. Забор крови через 3 и 4 часа также представляется обоснованным.

## ЗАКЛЮЧЕНИЕ

Таким образом, с целью дифференциальной диагностики псевдомальабсорбции и истинной мальабсорбции препаратов левотироксина натрия у пациентов с гипотиреозом мы рекомендуем проведение теста по оценке всасывания — однократный пероральный прием левотироксина натрия в дозе 600 мкг. Тест на всасывание левотироксина натрия является простым, безопасным и может проводиться в амбулаторных условиях. При интерпретации результатов теста в клинической практике рекомендуется оценка относительного прироста концентрации свТ4, выраженная в процентах. Всасывание левотироксина натрия при приеме внутрь может быть расценено как нормальное, если увеличение концентрации свТ4 хотя бы в одной из точек (через 2, 3 или 4 часа после приема болюсной дозы) составляет не менее 18%.

Оценка диагностической точности теста с определением его чувствительности и специфичности требует продолжения исследования: должен быть проведен анализ результатов в когорте пациентов с различными причинами декомпенсации гипотиреоза, в том числе с истинной мальабсорбцией.

## ДОПОЛНИТЕЛЬНАЯ ИНФОРМАЦИЯ

Источники финансирования. Работа выполнена в рамках государственного задания «Гормонально-метаболические и молекулярно-клеточные характеристики заболеваний щитовидной железы как основа для разработки инновационных методов диагностики, лечения и профилактики» (НИР № 123021300097-0).

Конфликт интересов. Авторы заявляют об отсутствии конфликта интересов.

Участие авторов. Все авторы одобрили финальную версию статьи перед публикацией, выразили согласие нести ответственность за все аспекты работы, подразумевающую надлежащее изучение и решение вопросов, связанных с точностью или добросовестностью любой части работы».
